# An Unusual Presentation of an Enterococcus faecalis Endocarditis With Wrist and Forearm Infection: A Case Report

**DOI:** 10.7759/cureus.57526

**Published:** 2024-04-03

**Authors:** Lia G Østerhegn, Kristina Procida, Emil L Fosbøl, Niels E Bruun

**Affiliations:** 1 Cardiology, University of Copenhagen, Copenhagen, DNK; 2 Cardiology, Zealand University Hospital, Roskilde, DNK; 3 Cardiology, Copenhagen University Hospital, Copenhagen, DNK

**Keywords:** an unusual case, duke criteria, non-cardiac focus, infective endocarditis, enterococcus faecalis

## Abstract

In this case, an unusual presentation of *Enterococcus faecalis* (*E.faecalis*) endocarditis and clinical signs of wrist and forearm infection are reported.

Before the patient was diagnosed with E.faecalis endocarditis, the patient managed to be treated with both prednisolone, various antibiotics, and colchicine on suspicion of gout, erysipelas, and deep tissue infection. Growth of *E.faecalis* in blood cultures raised the suspicion of endocarditis, and transesophageal echocardiography revealed vegetations on the aortic and the mitral valves with a perforation of the anterior mitral leaflet. Since the patient responded well to antibiotic treatment and there was no progression of the size of the vegetations or the perforation, it was decided by the endocarditis team to refrain from surgery.

*E. faecalis* endocarditis can be difficult to diagnose because the patients are often elderly, and symptoms may be subtle and misleading. In the present case, the diagnostic process was based on the Danish IE guidelines, which state that *E. faecalis* is a typical IE bacterium. Accepting *E. faecalis* as a typical infective endocarditis bacterium may lead to an earlier diagnosis and treatment.

## Introduction

Infective endocarditis (IE) is a lethal disease with an in-hospital mortality of 15-20% and a one-year mortality of 30-40% [1,2]. There is an increasing prevalence among patients with healthcare exposure, and around 10-20% of all cases of endocarditis in Denmark are now due to Enterococcus faecalis (E. faecalis) [3]. The typical presentation of a patient with E. faecalis endocarditis is a subacute case with fever, malaise, weight loss, and often a heart murmur [4].

IE is defined as an infection/inflammation of the inner lining of the heart or heart valves [5]. It is primarily a disease caused by bacteria, but rarely fungi constitute the infectious microorganism. Endocarditis is classified according to the valve involved and the timing of symptoms, either as an acute illness or, more often, as a subacute disease with symptoms lasting from weeks to several months [6]. The acute presentation is aggressive and often characterized by acute heart failure due to heart-valve destruction, sepsis, or systemic embolism [7]. In contrast, the subacute presentation is characterized by a diversity of unspecific symptoms, which makes IE challenging to diagnose. Across the last decades, E. faecalis has evolved as a more frequent cause of IE, particularly among elderly people with healthcare-associated infections [4,8]. The entrance of E. faecalis is often the urinary tract or the gastrointestinal tract, and symptoms of E. faecalis bacteremia leading to IE can be vague, obscuring the diagnosis [4]. Here, we present an insidious case of a patient where blood cultures with the growth of E. faecalis were the only essential lead to the diagnosis of subacute native valve endocarditis.

## Case presentation

**Figure 1:**
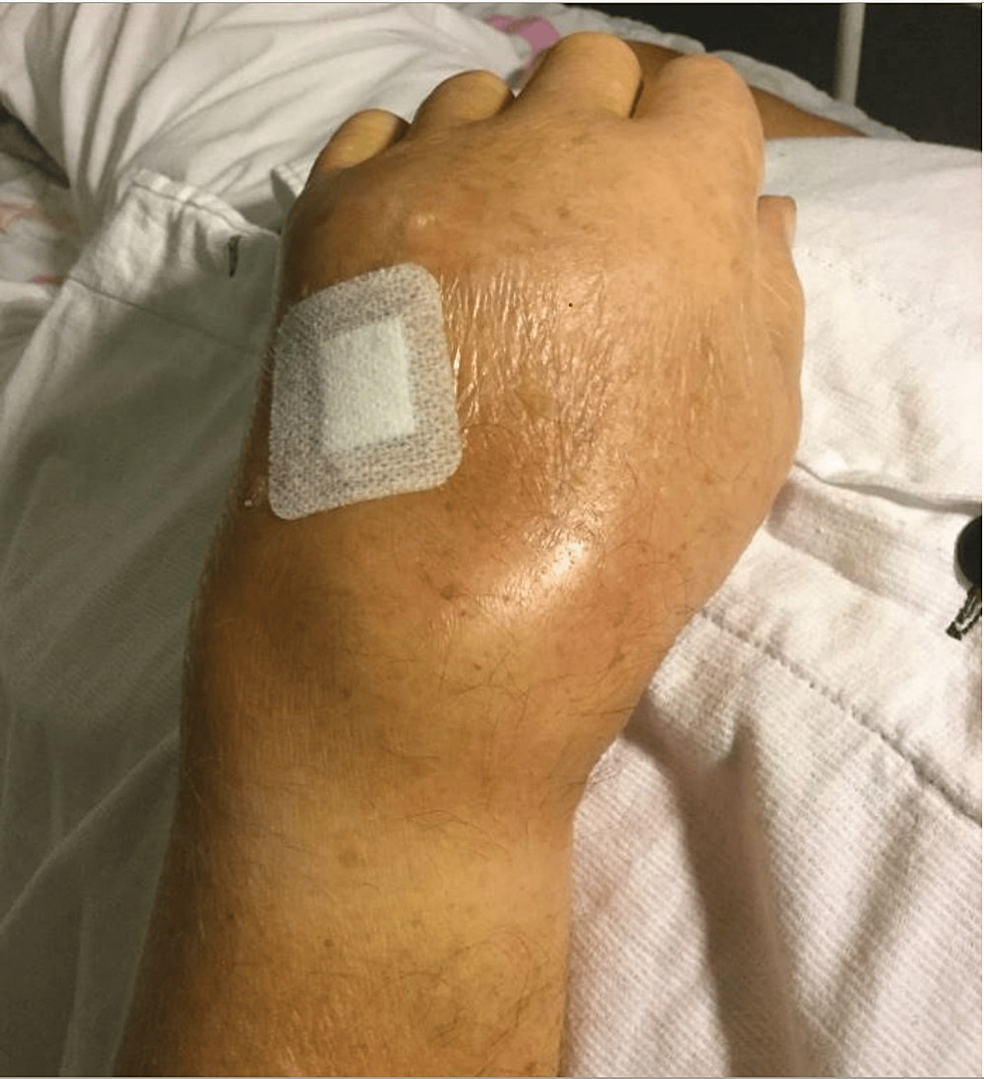
Left wrist as presented at the admission with redness and swelling on the dorsal side.

A 78-year-old Caucasian male was admitted with a medical history of hypertension, hypercholesterolemia, episodes of gout, cerebral apoplexy, coronary artery bypass graft surgery, and a dual chamber pacemaker. The patient consulted the General Practitioner (GP) after experiencing pain and swelling in his left hand and wrist for over a week. Since C-reactive protein (CRP) was increased to 120 mg/L (reference: < 8 mg/L), the GP started dicloxacillin and prednisolone treatment. However, the patient arrived at the local hospital's emergency department five days later because of continuing symptoms. The patient was afebrile at this examination, but the wrist was red and swollen, and the pain extended to the forearm (Figure 1).

The laboratory investigation showed leukocytosis of 19.7 X 109/L (reference: 3.5-8.8x10^9^/L) with neutrophilia and a CRP of 98 mg/L (reference: <8 mg/L). Phenoxymethyl penicillin was added based on the tentative diagnosis: Erysipelas. However, the patient did not feel well; the following day, he contacted the GP again.

The GP referred the patient to the emergency department because the symptoms had progressed to wrists and hands. The CRP and leukocytosis had both increased further to CRP 170 mg/L (reference: <8 mg/L) and 26.2x10^9^/L (reference: 3.5-8.8x10^9^/L). A combination of deep tissue infection and a gout attack was suspected. Therefore, the patient was admitted for intravenous treatment with benzylpenicillin and cloxacillin combined with oral treatment of prednisolone and colchicine. 

The next day, an ultrasound and an X-ray of the hand indicated synovitis and tendosynovitis with no sign of ostitis, which the rheumatologist inferred as arthritis urica. The prednisolone dose was increased, allopurinol was added, colchicine was continued, and the antibiotic treatment was stopped. 

However, blood cultures were drawn when re-admittance showed positive gram-positive cocci growth in 2 out of 3 cultures. Antibiotic treatment with intravenous benzylpenicillin and cloxacillin was restarted. 

**Figure 2:**
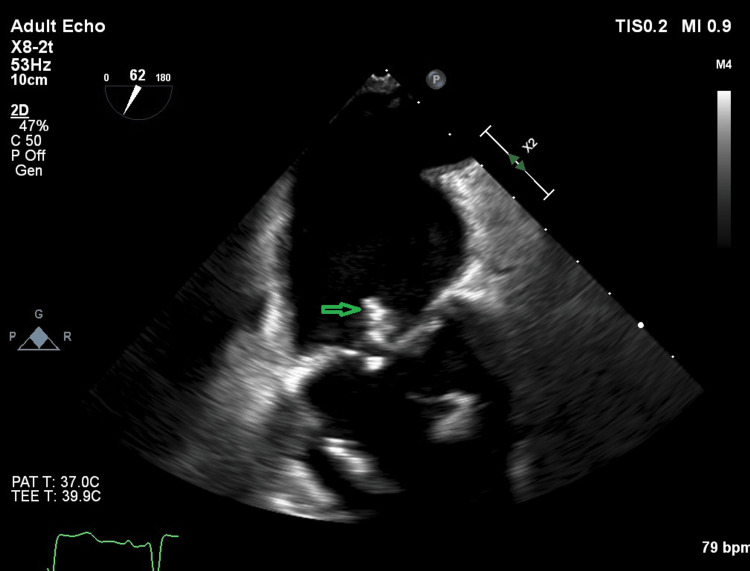
A transesophageal echocardiography of the patient with a vegetation of the mitral valve (arrow).

**Figure 3:**
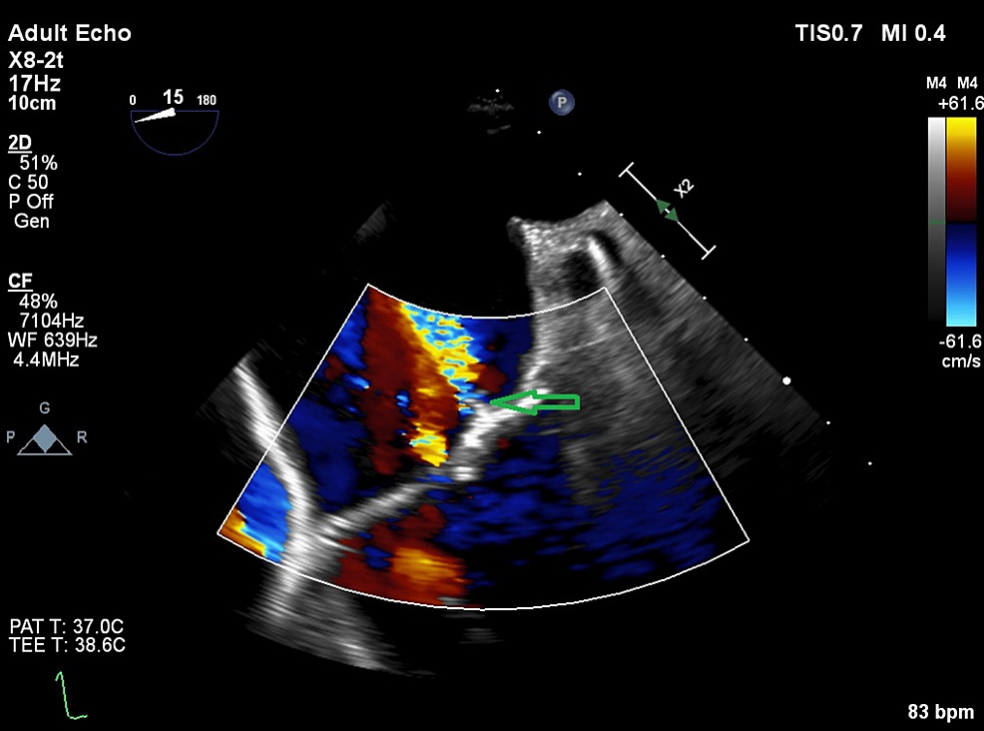
A transesophageal echocardiography of the patient with a perforation of the mitral valve (arrow).

**Figure 4:**
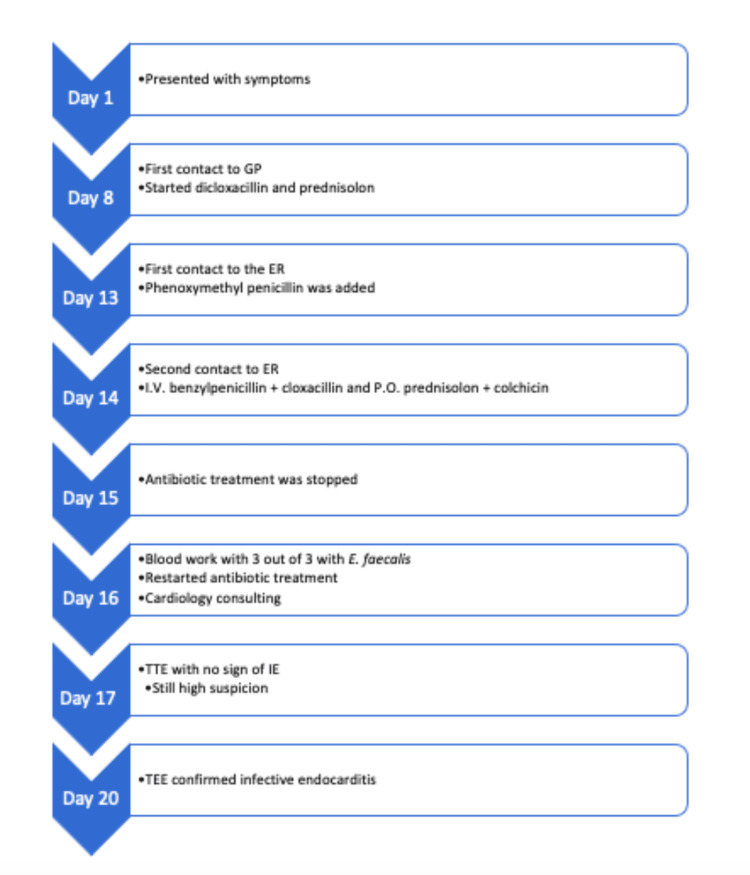
Timeline of the symptoms, initial investigations and treatment ER: Emergency room; TTE: Transthoracic echocardiography; TEE: Transesophageal echocardiography

The final results of the blood cultures revealed that 3 out of 3 cultures were positive for Enterococcus faecalis. The bacteria were sensitive to ampicillin, vancomycin, amoxicillin, and linezolid but resistant to cefuroxime. After consulting with the microbiology department, the antibiotic treatment was changed to ampicillin with cloxacillin, and the microbiologist suggested a cardiology assessment according to national guidelines [5]. A transthoracic echocardiography (TTE) was performed, but no definite signs of endocarditis were revealed. The left ventricle was not dilated, and the left ventricular ejection fraction was >60%. The aortic valve was calcified with a mild stenosis. In addition, a mild mitral insufficiency was present, the left atrium was not dilated, and the gradient across the tricuspid valve was normal. No definite signs of vegetation were seen on the heart valves or the pacemaker leads. However, suspicion of IE remained high, and a supplementary transesophageal echocardiography (TEE) showed an 8mm vegetation on the non-coronary cusp of the aortic valve with a discrete insufficiency but no abscess formation. In addition, the aortic valve's non-coronary cusp and right cusp were calcified. The mitral valve had a thickened edge on the anterior leaflet, which was suspected to be vegetation, and a 12 mm vegetation and a small perforation on the posterior leaflet (Figures 2-3). There was no vegetation on the pacemaker lead tricuspid- or pulmonary valves. The timeline of the symptoms, initial investigations, and treatments (Figure 4).

**Figure 5:**
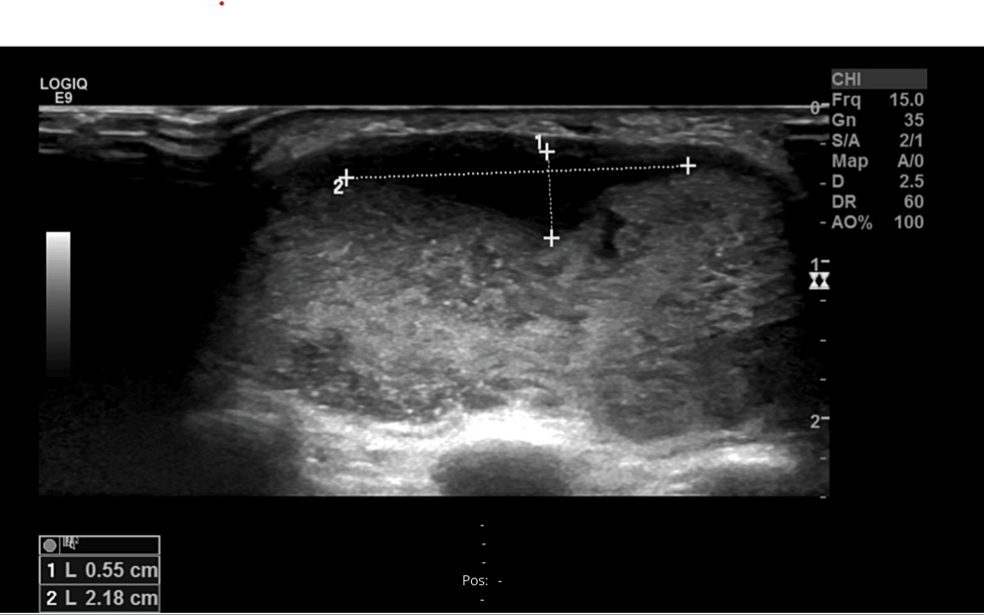
An ultrasound of the dorsal side of the left wrist. The lines show that the accumulation, which contained an inhomogeneous liquid, measured 2 cm X 0.5 cm X 6.5 cm

**Figure 6:**
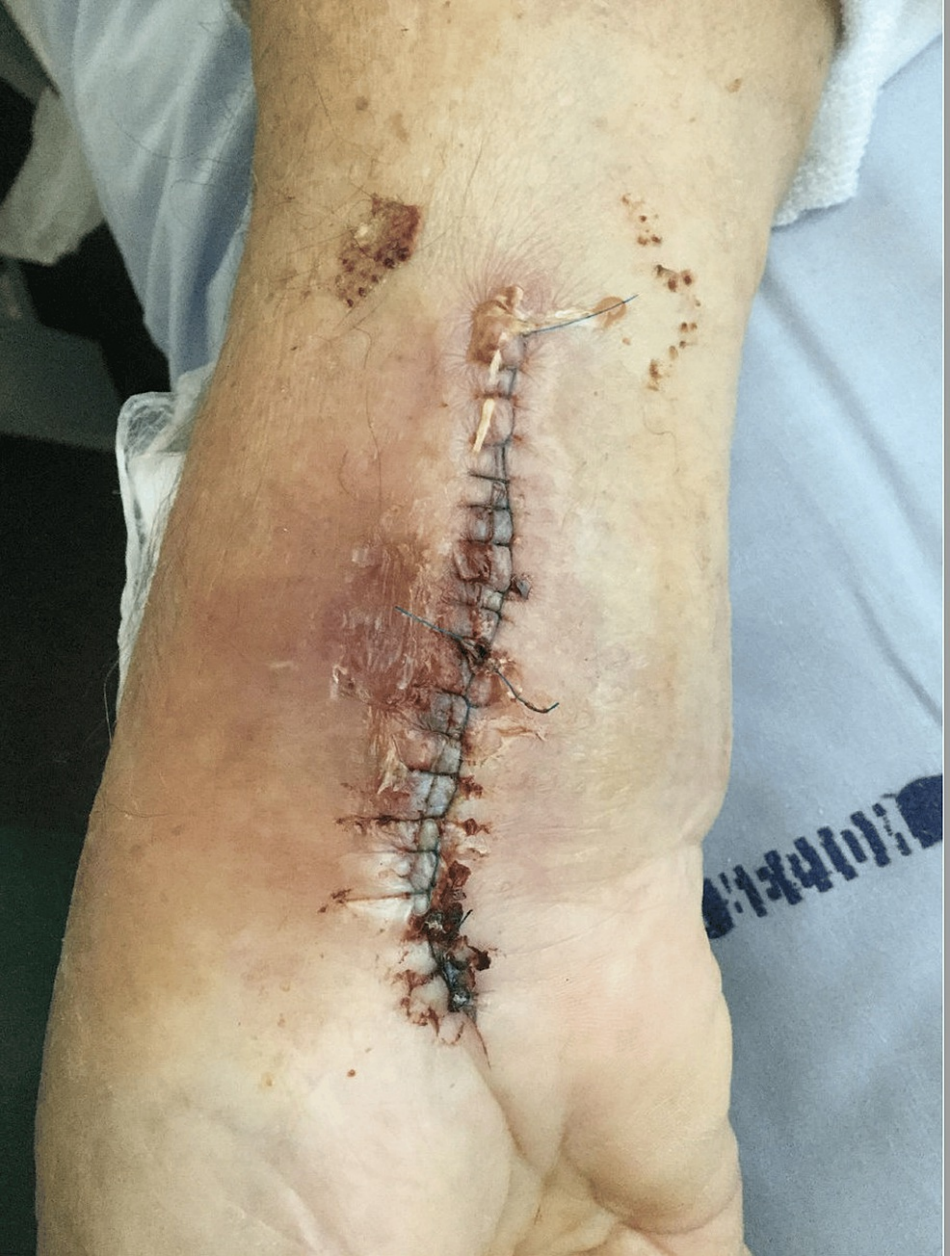
The patient's volar side on the right hand after surgical intervention with draining and cleaning the abscess.

**Figure 7:**
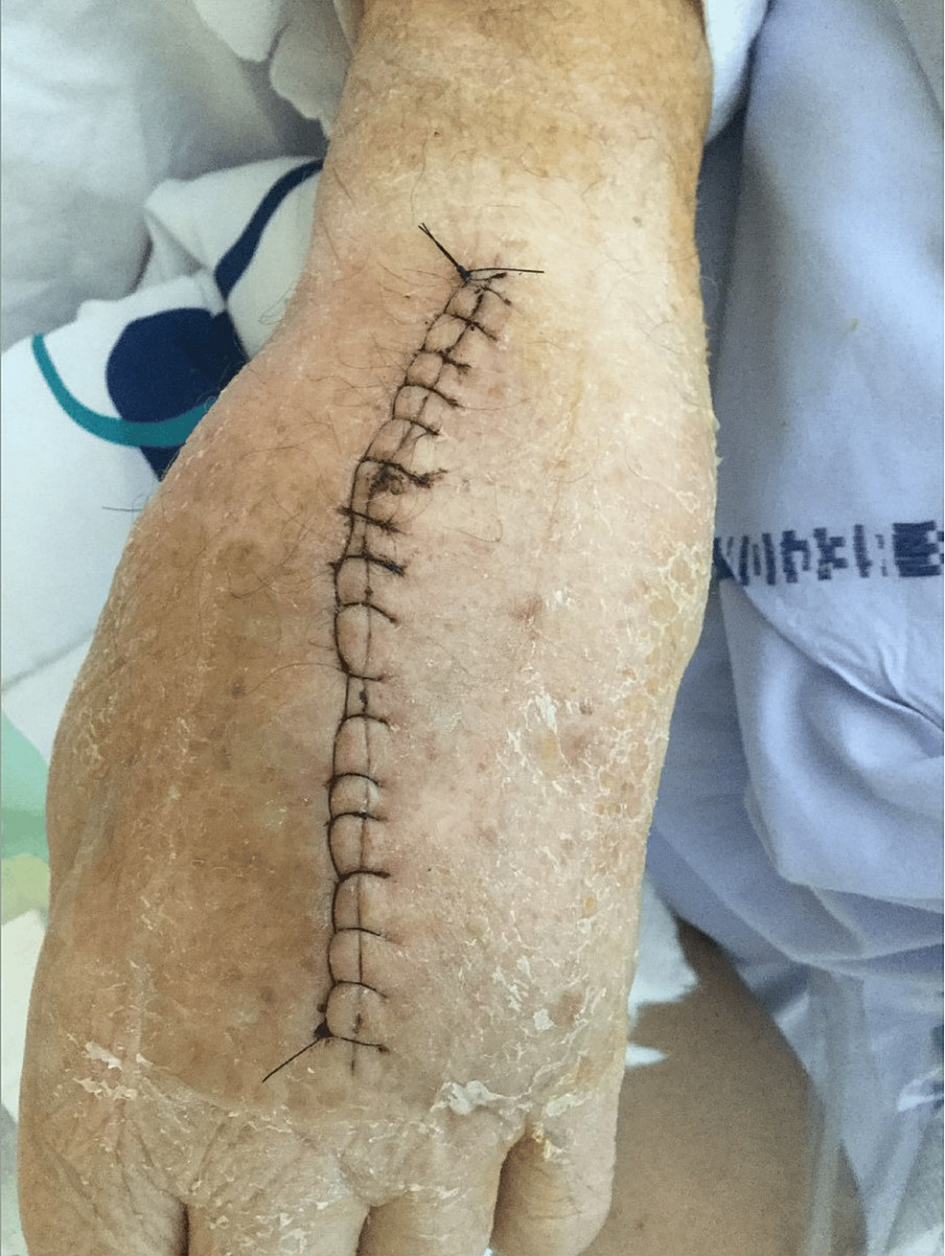
The patient's dorsal side of the left hand after surgical intervention with draining and cleaning the abscess.

Antibiotic treatment was adjusted in accordance with the IE diagnosis. Within one week, the TEE was repeated to evaluate if any changes in the valvular destruction or vegetation size had occurred. The perforation of the anterior mitral valve leaflet had not increased, and the size of the vegetation was unchanged. Subsequently, the patient was discussed with the endocarditis team, including microbiologists, a cardiologist, and a heart surgeon. Since valvular pathology was unchanged, they decided to continue medical treatment, and additionally, the patient was considered too frail for heart valve surgery. The wrists were also re-evaluated, and ultrasound revealed round cavities consistent with abscess formation (Figure 5). The abscesses on both hands were surgical cleft and drained for a purulent fluid and surgical cleansed (Figures 6-7).

**Figure 8:**
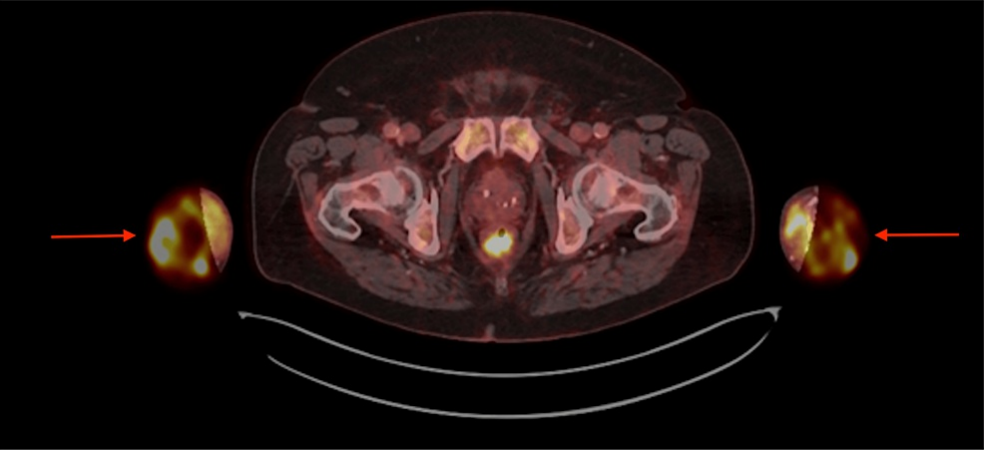
F-18-FDG PET/CT scanning with contrast uptake from both wrists (arrows) F-18-FDG: Flour-18-Fluorodeoxyglucose; PET/CT: positron emission tomography/ computed tomography

The fluid tested positive for E. faecalis growth. The pacemaker was extracted without any complications. A positron emission tomography/ computed tomography (PET/CT) performed after the pacemaker was extracted revealed positive Flour-18-Fluorodeoxyglucose (F-18-FDG) signals corresponding to both wrists and forearms and colon diverticulitis, indicating the primary portal of the entrance (Figure 8).

Subsequently, a coloscopy confirmed the presence of diverticula without malignancy. The patient underwent a six-week antibiotic treatment for the IE. In the end, TEE was repeated, and the perforation was unchanged. No significant arrhythmias were detected, and pacemaker reimplantation was not indicated. At one-year follow-up, the patient was in good condition and had not incurred a reinfection, and the clinical and echocardiographic conditions remained stable.

## Discussion

In this case, we present a patient with subacute native valve E. faecalis infective endocarditis (IE) and the unusual onset of symptoms in wrist and forearm infection.

Subacute IE is characterized by a fever >37.5°C and symptoms of poor appetite, weight loss, headache, and muscle pain or chills [9]. Around 20-40% of IE patients have embolic complications at the time of diagnosis [9]. This 78-year-old male presented with red and swollen wrists and forearms and none of the symptoms mentioned above. The patient was known to have gout, and the symptoms pointed the physicians in that direction. The only finding leading to the endocarditis diagnosis was the positive blood cultures with E. faecalis, underscoring the importance of up-front blood cultures in patients admitted with signs of infection. The entry focus of E. faecalis bacteremia is typically located in the gastrointestinal channel or the urinary tract [4], and most likely, the abscesses in the wrist and forearms were the result of hematogenous dispersion of the bacteria from the colon infection.

In Denmark, positive blood cultures with E. faecalis routinely lead the microbiologist to call clinicians to inform them of the risk of IE [5]. This warning system often leads to an earlier diagnosis and onset of treatment of endocarditis, decreasing the risk of complications and severe clinical outcomes [6]. The patient had a perforated mitral valve leaflet and a pacemaker in this case. However, the IE team concluded that according to the European Society of Cardiology (ESC) and national guidelines, the patient did not fulfill a class I indication for heart valve surgery. Further, frailty was high [10]. However, it was decided to explant the pacemaker by the European Heart Rhythm Association (EHRA) recommendations [11]. The indication for pacemaker implantation had been sick sinus syndrome with spells of dizziness. Since no significant arrhythmias were detected after the pacemaker was extracted and the patient was asymptomatic, it was decided to refrain from reimplantation. The patient has been followed for over one year with no signs of significant arrhythmia, further valve destruction, or reinfection.

In patients with E. faecalis bacteremia, a prevalence of IE above 20% has been demonstrated [12]. Consequently, the IE guidelines from the Danish Society of Cardiology consider E. faecalis a typical IE bacteria, and routine cardiology assessment is recommended [5,12]. Additional findings from imaging techniques are required to diagnose IE, particularly echocardiography. However, the implication of the present case on clinical practice is to underscore the need for heightened awareness of atypical presentations of IE. According to the modified Duke criteria published in 2000, one of the major criteria of IE requires Enterococcus bacteremia to be both community-acquired and without a primary focus [13]. However, only in around 40% of IE cases caused by E. faecalis, the bacteremia is community-acquired, and less than half of the cases have an absence of a primary focus [2]. A recent study in patients with E. faecalis bacteremia found that the 2000 Duke criteria have a sensitivity at 70% for the IE diagnosis [3]. If the Duke criteria were changed to accept E. faecalis as an IE bacterium per se, the sensitivity would increase to 96% [3]. By refraining from the 2000 Duke-mentioned criteria for E. faecalis in the updated Duke criteria, IE will be considered in an increasing number of patients with E. faecalis bacteremia. However, E. faecalis bacteremia should not be taken as an automatic IE indicator. However, we have previously shown that the risk of IE in patients with E. faecalis bacteremia increases significantly if one or more of the six risk factors observed in our prospective study (prosthetic heart valve, community acquisition, ≥3 positive blood culture bottles, uncertain portal of entry, monomicrobial bacteremia or immunosuppression) are present [2]. Therefore, a reason to await echocardiography in patients with E. faecalis bacteremia could be patients presenting with a UG or GI focus and none of these risk factors.

Most recently, the International Society of Cardiovascular Infectious Disease updated the Modified Duke criteria to a 2023 edition, and E. faecalis is now considered a typical IE bacterium [14]. The requirements for community acquisition and the lack of a primary focus have been omitted. Therefore, E. faecalis bacteremia per se should raise suspicion about IE. This change will hopefully result in patients with E. faecalis IE being diagnosed more quickly and treated correctly. The present case underscores that even if a patient presents with symptoms suggesting a non-cardiac focus of infection but blood cultures grow E. faecalis, the diagnosis of IE should readily be considered.

## Conclusions

In conclusion, Enterococcus faecalis endocarditis can be difficult to diagnose for patients with unusual symptoms. A blood culture with E. faecalis was the key to getting the right diagnosis for a patient with a non-cardiac focus. In 2023, E. faecalis was included as a typical IE bacterium in Duke criteria, hopefully leading to quicker diagnoses and treatment.

## References

[1] Habib G, Lancellotti P, Antunes MJ (2015). 2015 ESC guidelines for the management of infective endocarditis: the task force for the management of infective endocarditis of the European Society of Cardiology (ESC). Endorsed by: European Association for Cardio-Thoracic Surgery (EACTS), the European Association of Nuclear Medicine (EANM). Eur Heart J.

[2] Dahl A, Iversen K, Tonder N (2019). Prevalence of infective endocarditis in Enterococcus faecalis bacteremia. J Am Coll Cardiol.

[3] Dahl A, Fowler VG, Miro JM, Bruun NE (2022). Sign of the times: Updating infective endocarditis diagnostic criteria to recognize Enterococcus faecalis as a typical endocarditis bacterium. Clin Infect Dis.

[4] Dahl A, Bruun NE (2013). Enterococcus faecalis infective endocarditis: Focus on clinical aspects. Expert Rev Cardiovasc Ther.

[5] Moser C, Fosbøl EL, Rosenvinge F (2023). Infektiøs endokarditis. Dansk Cardiologisk Selskab.

[6] Cahill TJ, Baddour LM, Habib G (2017). Challenges in infective endocarditis. J Am Coll Cardiol.

[7] Ferrera C, Vilacosta I, Fernández C (2018). Early surgery for acute-onset infective endocarditis. Eur J Cardiothorac Surg.

[8] Østergaard L, Voldstedlund M, Bruun NE (2022). Temporal changes, patient characteristics, and mortality, according to microbiological cause of infective endocarditis: A nationwide study. J Am Heart Assoc.

[9] Sadeghpour A, Alizadehasl A (2022). Infective endocarditis. Practical Cardiology.

[10] Delgado V, Ajmone Marsan N, de Waha S (2023). 2023 ESC Guidelines for the management of endocarditis. Eur Heart J.

[11] Blomström-Lundqvist C, Traykov V, Erba PA (2020). European Heart Rhythm Association (EHRA) international consensus document on how to prevent, diagnose, and treat cardiac implantable electronic device infections-endorsed by the Heart Rhythm Society (HRS), the Asia Pacific Heart Rhythm Society (APHRS), The Latin American Heart Rhythm Society (Lahrs), International Society for Cardiovascular Infectious Diseases (ISCVID) and the European Society of Clinical Microbiology and Infectious Diseases (ESCMID) in collaboration with the European Association for Cardio-Thoracic Surgery (EACTS). Europace.

[12] Andersen MH, Holle SL, Klein CF (2020). Risk for infective endocarditis in bacteremia with Gram positive cocci. Infection.

[13] Li JS, Sexton DJ, Mick N (2000). Proposed modifications to the Duke criteria for the diagnosis of infective endocarditis. Clin Infect Dis.

[14] Fowler VG, Durack DT, Selton-Suty C (2023). The 2023 Duke-International Society for Cardiovascular Infectious diseases criteria for infective endocarditis: Updating the modified Duke criteria. Clin Infect Dis.

